# Cannabis and Rheumatoid Arthritis: A Scoping Review Evaluating the Benefits, Risks, and Future Research Directions

**DOI:** 10.5041/RMMJ.10509

**Published:** 2023-10-29

**Authors:** Nicole Paland, Haya Hamza, Antonina Pechkovsky, Miran Aswad, Dayana Shagidov, Igal Louria-Hayon

**Affiliations:** 1 Medical Cannabis Research and Innovation Center, Rambam Health Care Campus, Haifa, Israel; 2 Clinical Research Institute at Rambam (CRIR), Rambam Health Care Campus, Haifa, Israel

**Keywords:** Autoimmune disease, cannabis, CBD, inflammation, rheumatoid arthritis, THC

## Abstract

Rheumatoid diseases, including rheumatoid arthritis, osteoarthritis, and fibromyalgia, are characterized by progressive inflammation in the musculoskeletal system, predominantly affecting the joints and leading to cartilage and bone damage. The resulting pain and ongoing degradation of the musculoskeletal system contribute to reduced physical activity, ultimately impacting quality of life and imposing a substantial socioeconomic burden. Unfortunately, current therapeutics have limited efficacy in slowing disease progression and managing pain. Thus, the development of novel and alternative therapies is imperative. Cannabinoids possess beneficial properties as potential treatments for rheumatoid diseases due to their anti-inflammatory and analgesic properties. Preclinical studies have demonstrated promising results in halting disease progression and relieving pain. However, there is a scarcity of patient clinical studies, and the available data show mixed results. Consequently, there are currently no established clinical recommendations regarding the utilization of cannabis for treating rheumatoid diseases. In this review, we aim to explore the concept of cannabis use for rheumatoid diseases, including potential adverse effects. We will provide an overview of the data obtained from preclinical and clinical trials and from retrospective studies on the efficacy and safety of cannabis in the treatment of rheumatoid diseases.

## INTRODUCTION

Rheumatoid diseases are characterized by progressive chronic inflammation of the musculoskeletal system, afflicting primarily the joints but can lead to systemic comorbidities, such as pulmonary diseases or vasculitis. Chronic inflammation results in cartilage and bone damage, whose deterioration can lead to the disability of the affected patient.^1^

Rheumatoid diseases inflict a significant individual and societal burden. Pain and musculoskeletal deficits lead to a progressive decline in physical activity and quality of life and carry the risk of cumulative comorbidities.^2^ In addition, medical costs for treatment, as well as reduced work capacity and decreased societal participation of patients with rheumatoid arthritis (RA), have a significant socioeconomic effect on society.^3^

Treating rheumatoid disease is challenging not only because of its progressive nature but also because of the side effects of available therapies.^4^ Moreover, available treatment options cannot reverse rheumatoid diseases. Thus, therapy efforts are divided into preventive medicine (starting treatment before clinical manifestation) and developing new drugs. Three classes of drugs are currently available: (1) disease-modifying anti-rheumatic drugs (DMARDs), which target tumor necrosis factor (TNF)-α, the interleukin (IL)-6 receptor, and stimulate the depletion of T and B cells, thereby slowing the progression of the structural damage;^5^,^6^ (2) non-steroidal anti-inflammatory drugs, which improve physical function by reducing pain and stiffness, but do not modify disease progression;^4^ and (3) glucocorticosteroids, which have a rapid symptomatic and disease-modifying effect.^7^

Prolonged use of glucocorticoids and DMARDs has long-term severe adverse effects.^8^,^9^ Moreover, patients treated with biological DMARDs have an increased risk of severe infections by tuberculosis and herpes zoster virus, as well as an elevated risk of developing melanoma.^10^ This exemplifies the need for novel treatment approaches and safe therapeutics. One approach is medicinal cannabis use, which takes advantage of its pain-reducing and immune-modulating features.

## CANNABIS AND CANNABINOIDS

**Figure 1:**
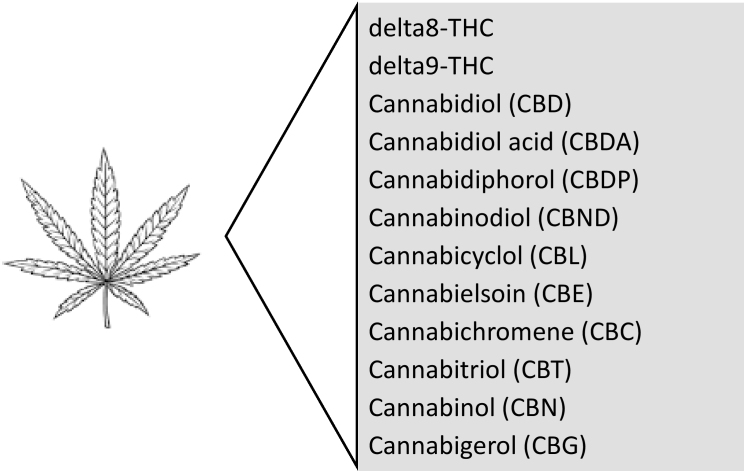
Phytocannabinoids: A Representative List Found in the Cannabis Plant.

Cannabis is the most widely used illicit drug in the world. Cannabis is not a single substance but consists of more than 550 different chemical constituents accumulated in the cannabis plant, among them approximately 150 psychoactive and non-psychoactive cannabinoids and over 400 non-cannabinoids. The cannabis plant (*Cannabis sativa*) belongs to the Cannabaceae family. There are two major forms of *Cannabis sativa*: marijuana, which has high levels of the psychoactive tetrahydrocannabinol (THC); and hemp, which has high levels of non-psychoactive cannabinoids and low THC levels.^11^ The two main pharmacologically active THC compounds are Δ8-THC and Δ9-THC. The main non-psychoactive pharmacologically active cannabinoids include cannabinol, cannabidiol (CBD), and cannabigerol (Figure 1), as well as non-cannabinoids like flavonoids, terpenes, and fatty acids.^12^,^13^

Cannabinoids mediate their biological and therapeutic effects through the G-protein coupled receptors cannabinoid receptor 1 (CB1R) and 2 (CB2R).^14^–^16^ The G-proteins act as adaptors that link G-protein coupled receptors to intracellular signaling and regulatory proteins to activate or modulate signaling pathways. Other G-protein coupled receptors, such as GPR55 and GPR18, and transient receptor potential (TRP) channels, such as TRPV2, TRPA1, and TRPM8, are also involved in cannabinoid signaling.^17^ Highly expressed in the central nervous system, CB1R is found in particularly high levels in the neocortex, hippocampus, basal ganglia, cerebellum, and brainstem.^18^ Conversely, low expression levels are observed in the peripheral nervous system. The CB1R binds the main active ingredient of marijuana, Δ9-THC, and mediates most of the THC effects in the central nervous system.^19^ The CB2R is present at high levels in the immune system and is commonly associated with regulating immune function. Additionally, CB2R is also located in the brain,^20^ where it is primarily localized to microglia, the central nervous system resident macrophages.^21^

The fact that both CB1 and CB2 receptors are expressed by immune cells suggests that cannabinoids play an important role in immune system regulation. For example, cannabinoids have been shown to exert anti-inflammatory effects in various *in vivo* and *in vitro* experimental models. In addition, several studies have shown that cannabinoids downregulate cytokine and chemokine production and upregulate T-regulatory cells to suppress inflammatory responses.^16^,^22^

### Immunomodulatory Effects of Cannabinoids on Rheumatoid Disease Progression: Insights from Preclinical *In Vitro* Models of Cytokine Regulation

Several studies have reported that CBD reduces the formation of reactive oxygen species and nitric oxide in various cell lines and animal models of inflammation. In addition, CBD blocks production of TNFα, the pro-inflammatory cytokines IL-1β, IL-2, IL-6, and IL-8, and the transcription factor nuclear factor (NF)-κB.^16^,^23^ Furthermore, extract with high CBD content exhibits the remarkable ability to diminish cytokine secretion from T cells derived from human donors. Additionally, a specific strain with high CBD content (CBD-X) demonstrates a potent capacity to effectively suppress cytokine storm in a mouse model.^24^

More evidence of the ability of cannabinoids to modulate the immune system comes from *in vitro* studies that showed that Δ9-THC suppressed the cell-mediated T helper 1 (Th1) response and enhanced Th2-associated cytokine secretion.^25^ This response prevents the activation of inflammatory signaling pathways, such as the NF-κB,^26^ mitogen-activated protein kinase, and Janus kinase/signal transducer and activator of transcription (JAK/STAT) pathways in immune cells.^27^

Another study, using lipopolysaccharide-activated BV-2 microglial cells, reported that both THC and CBD decreased pro-inflammatory signaling activation by reducing the activation of the JAK/STAT pathway. Furthermore, CBD suppressed NF-κB pathway activity and potentiated an anti-inflammatory negative feedback loop via JAK/STAT3.^28^

A recent study demonstrated that CBD directly affects microRNA (miRNA) expression. Results showed that CBD downregulated miR146a expression, which acts as a negative regulator of inflammation, in both resting and lipopolysaccharide-stimulated cells, thereby contributing to CBD’s ability to downregulate pro-inflammatory cytokines. Additionally, CBD upregulated miR34a in BV-2 microglia cells, which has several roles in cell survival, such as cell cycle, apoptosis, and differentiation. These results suggest that CBD-induced alterations in miRNA expression are part of the mechanism by which CBD suppresses immune function.^29^

Similarly, synthetic cannabinoids HU-210 and WIN55,212-2 prevent IL-1α-induced matrix degradation in bovine chondrocytes *in vitro*. In addition, both cannabinoids inhibited IL-1α-induced proteoglycan breakdown and collagen degradation. More-over, WIN55,212-2 inhibits inducible nitric oxide synthase and COX-2 expression, as well as NF-κB activation.^30^ This effect was reproduced using the endocannabinoid anandamide and potentiated by the CB1 and CB2 antagonists AM281 and AM630.^32^

### Insights into Cannabinoid Efficacy in Rheumatoid Disease Progression Management from Animal Studies

Several murine rheumatoid disease animal models have been used to investigate the possible anti-rheumatic efficacy of cannabinoids. For example, Zurier et al. investigated the impact of orally administering dimethylheptyl-THC-11-oic acid (DMH-11C), a non-psychoactive precursor of THC, on acute inflammation and chronic polyarthritis in male Lewis rats. Acute inflammation was induced by subcutaneously injecting IL-1β or TNFα into pouches on rats’ backs. In this model, oral administration of DMH-11C reduced the number of polymorphonuclear leukocytes in the pouches 6 hours after inducing inflammation.^32^ Furthermore, chronic polyarthritis was induced by intradermal injection of Freund’s complete adjuvant (2 ng *Mycobacterium butyricum* in 0.1 mL mineral oil), which causes polyarthritis in all four paws. This adjuvant-induced chronic polyarthritis was prevented by DMH-11C.^32^

Similarly, tetrahydrocannabinolic acid and THC alleviate collagen-induced arthritis in mice via CB1 by preventing the infiltration of inflamed cells into the synovium, which reduces hyperplasia and cartilage damage.^33^ Moreover, nociception can be diminished by adding THC and the endocannabinoid anandamide to male Sprague-Dawley rats after adjuvant-induced arthritis. The CB1 receptor antagonist SR142716A blocks anti-nociception developed after administering THC but not anandamide, suggesting that anandamide signaling is not limited to the CB1 receptor pathway. However, the effects of THC and anandamide can be inhibited by naloxone, indicating that they induce the release of endogenous opioids that mediate the anti-nociceptive effect.^34^

Oral or intraperitoneal administration of CBD has an anti-arthritic effect in acute and chronic relapsing collagen-induced arthritis (CIA). In both methods, joints were protected against severe damage via reduced interferon γ production and TNFα release from knee synovial cells.^35^ In addition, THC induced a CB1-mediated anti-nociceptive, but not hyperalgesic, effect, as observed in an adjuvant-induced arthritis model in Sprague-Dawley rats.^36^

The synthetic non-psychoactive cannabinoid HU-320 has potent anti-inflammatory and immunosuppressive properties. These anti-arthritic effects were observed in a murine collagen-induced arthritis model. In addition, daily peritoneal administration of HU-320 significantly ameliorated CIA by protecting the paw joints from pathologic damage and suppressing TNFα secretion from macrophages in the serum.^37^

Transdermal CBD administration also reduces inflammation and pain-related behaviors in an adjuvant-induced arthritis model in Sprague-Dawley rats. Cannabidiol (CBD) gel, applied for four consecutive days on the afflicted joint, significantly reduced joint swelling and pro-inflammatory markers. The paw withdrawal latency to noxious heat stimulation recovered to near baseline, but exploratory behavior was not altered, suggesting that CBD had a limited impact on brain function. These results indicate that transdermal administration of CBD can exert long-lasting anti-arthritic effects achieved without neuronal side effects (summarized in Table 1).

**Table 1:** Preclinical Trials—Animal Studies.

Reference	Research Question/Goal	Induction of Arthritis	Experimental Approach	Outcome
Zurier et al. (1998)^32^	Evaluation of anti-inflammatory activities of a non-psychoactive synthetic derivative of THC (DMH-11C)	*Acute Inflammation:* Induction of acute inflammation in dorsal air pouches with injection of IL-1β and TNFα *Chronic polyarthritis*: Intradermal injection of Freund’s complete adjuvant (2 mg *Mycobacterium butyricum* in 0.1 mL mineral oil)	Pouches placed on backs of male Lewis rats, inflammation induced with intradermal Freund’s adjuvant, and arthritis visually assessed in all four paws. Lewis rats assigned to groups and treated 3 days after adjuvant Tx: untreated treated with safflower oil treated with 0.1 mg/kg DMH-11C three times a week for 35 days	Oral administration of DMH-11C: *Acute inflammation:* reduced accumulation of pouch-filled polymorphonuclear leukocytes *Chronic polyarthritis:* - diminished arthritis severity - prevented severe joint tissue injury - chronic polyarthritis rats remained active with no weight loss compared to arthritic control rats - developed only mild joint synovitis
Smith et al. (1998)^34^	Effectiveness of Δ9-THC and anandamide in blocking nociception or chronic inflammation	Intradermal injection of Freund’s complete adjuvant (2 mg *Mycobacterium butyricum* in 0.1 mL mineral oil)	Freund’s adjuvant was injected into the plantar aspect of the paw in male Sprague-Dawley rats	Intraperitoneal administration of Δ9-THC or anandamide: - anti-nociceptive effect of Δ9-THC and anandamide - SR141716A (CB1 inhibitor) blocked THC, but not anandamide-induced nociception - no potential contribution of endogenous anandamide in arthritis
Malfait et al. (2000)^35^	Anti-arthritic properties of non-psychoactive CBD	Murine CIA model in complete Freund’s adjuvant	Intradermal injection at tail base of male DBA/1 mice of CII emulsified in complete Freund’s adjuvant Animals assigned into two groups: Intraperitoneal administration at clinical signs onset for 10 days, at different concentrations 20 mg/kg (*n*=12), 10 mg/kg (*n*=17), 5 mg/kg (*n*=15), 2.5 mg/kg (*n*=9) dissolved in ethanol:cremophor:saline (1:1:18); or vehicle alone (*n*=23) Oral administration at clinical signs onset for 10 days, CBD dissolved in olive oil at 10 mg/kg, 25 mg/kg, and 50 mg/kg (*n*=6 per group); control mice fed olive oil (*n*=6)	Intraperitoneal or oral administration of CBD: - blocked progression of RA - protected joints from severe damage - diminished CII-specific proliferation of lymph nodes and IFNγ-production *ex vivo* - decreased release of TNFα by knee synovial cells
Sumariwalla et al. (2004)^37^	Anti-inflammatory and immunosuppressive effects of synthetic cannabinoid HU-320	Murine model of CIA	HU-320 or vehicle control (mixture of cremophor-EL, absolute ethanol, and PBS) administered peritoneally for 10 days to arthritic mice at different doses from day 1 of clinical signs appearance at 0.5, 1, and 2 mg/kg body weight	Daily intraperitoneal administration of HU-320 - ameliorated CII-induced arthritis - protected foot joints from pathological damage - down-regulated CII-specific and polyclonal responses of murine lymphocytes - suppressed serum TNFα levels
Cox et al. (2007)^36^	Anti-nociceptive effect of Δ9-THC	Freund’s adjuvant-induced arthritis in Sprague-Dawley rats	Male Sprague-Dawley rats injected intradermally at the tail base with 0.1 mL vehicle or Freund’s adjuvant - arthritic or non-arthritic rats intraperitoneally injected with SR144528 (CB2 inhibitor), SR141716A (CB1 inhibitor), or vehicle (1:1:18, emulphor:ethanol:saline) - arthritic and non-arthritic rats intraperitoneally injected with Δ9-THC - paw pressure test to evaluate nociception	Intraperitoneal administration of Δ9-THC: - anti-nociceptive effect - SR144528 attenuated anti-nociceptive effect in arthritic but not in non-arthritic rats - SR141716A attenuated anti-nociception in arthritic and in non-arthritic rats
Hammell et al. (2016)^38^	Anti-inflammatory and anti-nociceptive effects of CBD gels	Freund’s complete adjuvant-induced arthritis in Sprague-Dawley rats	Sprague-Dawley rats (*n*=54) assigned to three groups and treated as follows: non-treated (*n*=21) one knee joint injected with Freund’s complete adjuvant vehicle or gel containing 1% or 10% CBD administered to backs of each animal for four consecutive days after arthritis onset	Transdermal administering of CBD gels: - significantly reduced joint swelling - reduced immune cell infiltration and thickening of the synovial membrane - paw withdrawal latency recovered to near baseline - dose-dependent reduction of pro-inflammatory biomarkers - did not alter exploratory behavior
Palomares et al. (2020)^33^	Evaluation of anti-arthritis properties of Δ9-THCA-A	Murine model of CIA	7-week-old DBA/1 mice treated twice with 100 μL type II bovine collagen, injected intradermally at tail base Mice assigned to groups (*n*=9) and treated with: Δ9-THCA-A ± PPARγ inhibitor, ± CB1 receptor antagonist, or vehicle Paw edema evaluation on scale of 0–4	Intraperitoneal injection of Δ9-THCA-A prevents: - arthritis in CIA mice - infiltration of inflammatory cells - synovium hyperplasia - cartilage damage - the expression of inflammatory and catabolic genes in knee joints - anti-arthritic activity mediated by CB1 receptor and PPARγ pathway

CB1, cannabinoid 1; CBD, cannabidiol; CIA, collagen-induced arthritis; CII, type II collagen; DBA/1 mice, strain of mice, DMH-11C, dimethylheptyl-THC-11-oic acid; IFN, interferon; IL, interleukin; PBS, phosphate-buffered saline; PPAR, peroxisome proliferator-activated receptor; RA, rheumatoid arthritis; THC, tetrahydrocannabinol; THCA-A, tetrahydrocannabinolic acid; TNF, tumor necrosis factor; Tx, treatment.

### Cannabinoid Effect on Rheumatoid Disease Progression in Synovial Fluid

In 2007, Richardson et al. showed that the endocannabinoid system plays a role in rheumatoid diseases. The authors examined the synovial fluid of 32 patients with osteoarthritis (OA) and 13 with RA after total knee arthroplasty. The endocannabinoids 2-arachidonyl glycerol as well as cannabinoid receptors CB1 and CB2 mRNA and protein levels were found in the synovial fluid of OA and RA patients but not in healthy donors. Furthermore, receptor stimulation was correlated to the activation of extracellular signal-regulated kinase (ERK1/2), which was blocked by the CB1 antagonist SR141716A. These results suggest the involvement of the endocannabinoid system in the development of rheumatoid diseases.^39^

In RA, pro-inflammatory cytokines and matrix metalloproteinases (MMPs) are released into the synovial tissue, where they promote cartilage degradation and bone erosion, leading to bone deformities.^40^,^41^ *Ex vivo* experiments performed by Johnson et al. showed that ajulemic acid, a non-psychoactive cannabinoid acid, suppressed the production of MMPs from fibroblast-like synovial cells taken from the affected joints of patients suffering from OA, or RA, or psoriatic arthritis.^42^ Furthermore, the synthetic cannabinoids CP55,940 and WIN55,212-2 significantly reduced the secretion of the pro-inflammatory cytokines IL-6 and IL-8 from IL-1β-stimulated synovial fibroblasts extracted from patients with RA with knee joint involvement (OA) and knee joint replacement surgery. This study showed that this effect was not mediated by CB1 and CB2,^43^ suggesting the involvement of other cannabis-related receptors.^43^ Another group confirmed the WIN55,212-2 results and further showed that treatment reduced the release of the MMP3 from synovial fibroblasts from RA and OA patients. This effect was mediated by transient receptor potential cation channel (TRP) subfamily V1 (TRPV1) and TRP subfamily A1 (TRPA1), not CB1 and CB2.^44^ A follow-up study showed that CBD also reduces the secretion of IL-6, IL-8, and MMP3 from synovial fibroblasts from RA patients. Furthermore, CBD increased intracellular calcium levels and reduced cell viability via TRPA1 but not TRPV1. Moreover, blocking the mitochondrial permeability transition pore by cyclosporine A prevented the CBD effects on cell viability and IL-8 production. Additionally, CBD’s effects were enhanced by adding TNFα, suggesting that CBD preferably acts in a pro-inflammatory environment and that CBD might ameliorate arthritis by targeting pro-inflammatory synovial fibroblasts.^45^

In the same year, another group showed in a 4-week, randomized, placebo-controlled, double-blinded study in a spontaneous canine model of OA that CBD, administered as naked CBD or liposomal-encapsulated CBD, could inhibit the production of pro-inflammatory cytokines IL-6 and TNFα while also increasing the anti-inflammatory cytokine IL-10. Alongside, the pain was significantly decreased, which led to a dose-dependent increase in mobility. Interestingly, naked CBD required a higher dosage (50 mg/day) for the same effect as 20 mg/day of liposomal CBD. These results point to the safe therapeutic potential of cannabinoids for alleviating pain.^46^

In accordance with these studies, we published similar results using high-THC or high-CBD extracts in mouse models of systemic or local lung inflammation. High-CBD, but not high-THC, attenuated the pro-inflammatory cytokines IL-1β and TNFα levels alongside a concomitant increase in the anti-inflammatory cytokine IL-10. Moreover, we observed that the migration of inflammatory neutrophils to the site of infection was decreased by the high-CBD extract, resulting in reduced levels of the pro-inflammatory cytokines IL-1β, MCP-1, IL-6, and TNFα in the inflamed lung. However, of the three tested high-CBD extracts, only one showed these inhibitory effects, explaining why studies on the influence of cannabinoids show ambiguous results. More research is needed into this phenomenon, including clinical studies on humans with extracts that showed therapeutic effects in previous animal studies.^24^

In contrast to the anti-arthritic properties observed in animal and *ex vivo* studies, Kotschenreuther et al. observed an increase in the differentiation of pro-inflammatory Th17 T-helper cells isolated from the peripheral blood of patients with rheumatoid or psoriatic arthritis or systemic lupus erythematosus treated with CBD oil or the endogenous cannabinoid anandamide for 4–8 weeks. The authors argue that the variability of CBD receptors between animal models and humans could contribute to the discrepancies. Moreover, many animal studies use CB1 or CB2 inhibitors to investigate the function of cannabinoids. Therefore, the authors suggest using cannabinoids in RA patients with caution (summarized in Table 2).^47^

**Table 2:** Studies with Synovial Fluid.

Reference	Research Goal	Study Design	Intervention	Participants, *n* (F/M)	Cannabis Application	TX Duration	Efficacy
Johnson et al. (2007)^42^	AjA influence on MMP production in human FLS and role of PPAR□	*Ex vivo* study on FLS from RA pts	AjA	Synovial fluid extracted from joints of RA, OA, or psoriatic arthritis pts	AjA acid in DMSO	60 min AjA Tx followed by stimulation with 10 ng/mL rhIL-1α rhTNFα for 18–24 hours	- AjA suppressed MMP production from FLS, independent of PPAR□ - AjA suppressed MMP3 secretion in TNFα- and IL-1α-stimulated PPAR^+/-^ and PPAR^-/-^ MEFs
Richardson et al. (2008)^39^	Determine if cannabinoid signaling elements are present in synovia of RA or OA pts	*Ex vivo* synovial fluid	Endocannabinoid levels were quantified in synovial biopsies	OA (*n*=32) or RA (*n*=13) pts undergoing knee arthroplasty and healthy volunteers	No	No	- CB1 and CB2 protein and RNA were present in synovia of OA and RA pts - Cannabinoid receptor stimulation time-dependently activated MAPK/ERK1/2, which is blocked by SR141716A (CB1 antagonist) - AEA and 2-AG found in synovia of OA and RA pts but not healthy volunteers - Fatty acid amide hydrolase active in synovia of OA and RA pts
Selvi et al. (2008)^43^	Anti-inflammatory properties of synthetic cannabinoids CP55,940 and WIN	*Ex vivo* study on FLS from pts with RA	Synthetic cannabinoids CP55,940 and WIN	Synovial fluid from 5 pts with RA and knee joint involvement (OA knee joint replacement surgery)	FLS treated with CP55,940 and WIN for 3 hours Measurement of pro-inflammatory cytokines before and after Tx	3 hours 1-hour pre-Tx with cannabinoid receptor antagonist, 1-hour pre-incubation or with 0.1 ng/mL IL-1α	- Both cannabinoids induced potent and significant reduction of IL-6 and IL-8 secretion from IL-1α-stimulated FLS - Independent from antagonistic actions
Lowin et al. (2016)^44^	Anti-arthritic properties of synthetic cannabinoid WIN	*Ex vivo* study of RASF pts	CBD	*n*=21/7 Longstanding RA Mean age 61.1±10.7	CBD in DMSO	Up to 72 min	- Concentration-dependent reduction/inhibition of IL-6, IL-8, and MMP3 secretion - High WIN concentration effects partly dependent on media FCS content - WIN influences adhesion to fibronectin (low concentrations <2 μM stimulated adhesion; higher concentrations >2 μM decreased adhesion) - WIN concentrations >1 μM halted cell proliferation - WIN at high concentrations >2 μM changed cell shapes from spread out with fibropodia to sharp-edged with a condensed nucleus and extensive vacuolation - WIN activities were independent of CB1 or CB2 but attenuated by TRPV1 or TRPA1 inhibitors
Lowin et al. (2020)^45^	Evaluation of anti-arthritic properties of CBD	Investigation of RASF *ex vivo* from RA pts		32 F and 8 M with long-standing RA fulfilling ACR criteria, mean age 66.9±8.2 years			- CBD reduced cell viability and proliferation of RASF - CBD increased intracellular Ca^2+^, effects enhanced by TNFα - Ca^2+^ mobilization and PoPo3 uptake partly depended on TRPA1 activation - Mitochondrial targets mediate CBD effects
Kotschenreuther et al. (2021)^47^	Analysis of cannabinoid impact on Th17 differentiation in RA pts	Investigation of T helper cells differentiation *ex vivo* in peripheral blood cells of RA pts		Healthy donors	RA pts		- CBD and AEA increased Th17 differentiation in CD4^+^ T cells of RA pts but not healthy donors - Adding cytokines TGFβ, IL-1α, IL-6, and IL-23 increased Th17-inducing CBD properties - CBD oil Tx for 4–8 weeks increased Th17 cell expansion - Disease Activity Score 28-joint count C-reactive protein increased during CBD Tx - Immunomodulatory effect of CBD not mediated by CB1, CB2, or GPR55 - CBD increased *sgk1* and *ahr* expression and decreased *csf2* expression - Results contrary to results in mice where CBD ameliorated RA

2-AG, 2-arachidonyl glycerol; ACR, American College of Rheumatology; AEA, N-arachidonoylethanolamine; AjA, ajulemic acid; CB1/CB2, cannabinoid 1/cannabinoid 2; CBD, cannabidiol; DMSO, dimethylsulfoxide; F, females; FCS, fetal calf serum; FLS, fibroblast-like synovial cells; GPR55, G protein-coupled receptor 55; M, males; MAPK/ERK1/2, mitogen-activated protein kinase/extracellular-regulated kinase; MEFs, mouse embyonic fibroblasts; MMP, matrix metalloproteinase; OA, osteoarthritis; PPAR, peroxisome proliferator-activated receptor; pts, patients; RA, rheumatoid arthritis; RASF, rheumatoid arthritis synovial fibroblasts; rh, recombinant human; RNA, ribonucleic acid; TNF, tumor necrosis factor; Th17, T helper 17; TRPA1, transient receptor potential cation channel subfamily A1; TRPV1, transient receptor potential cation channel subfamily V1; Tx, treatment; WIN, WIN55,212-2.

### Effects of Medicinal Cannabis on Rheumatoid Pain

Rheumatoid disease is characterized by chronic pain, which significantly decreases the quality of life of those afflicted. Currently, efficacious treatment and adequate pain management are unavailable for rheumatoid diseases. Thus, alternative therapies for pain management are needed. The impact of medicinal cannabis extracts on chronic pain has been evaluated in several randomized, double-blind, placebo-controlled clinical trials. For example, Notcutt et al. compared three cannabis-based medicinal extracts containing THC, CBD, or a mixture, on 34 patients for 12 weeks. The THC-based extracts were most effective in pain control when used as a sublingual spray, with only mild side effects.^48^ Another group tested nabiximols (Sativex®) which comprises an even combination of CBD and THC (each 100 microlitres contains 2.7 mg THC and 2.5 mg CBD) on 58 RA patients. Over five weeks, Sativex® was administered as an oromucosal spray in the evening. Patients were evaluated for movement and resting pain, morning stiffness, and sleep quality using the Short Form McGill Pain Questionnaire and the DAS28 measure of disease activity. Statistically significant improvements with Sativex® alleviated movement and resting pain as well as sleep quality, but not morning stiffness. In addition, no signs of withdrawal and severe side effects were observed.^49^

Other studies tested the analgesic effects of cannabis in patients with neuropathic pain when administered via a vaporizer. In one double-blind, placebo-controlled crossover study, 35 patients with central and peripheral neuropathic pain received THC-based cannabis medium-dose (3.53%) or low-dose (1.29%), or placebo. As measured by the pain intensity score of a visual analogue scale, the analgesic response showed an effect similar to efficacies obtained by conventional pain relievers. Only mild reversible psychoactive effects of limited time duration were measured.^50^ Another recent randomized, placebo-controlled four-way crossover trial investigated the analgesic effects of inhaled pharmaceutical-grade cannabis. Four different cannabis variants with known THC and CBD content were tested on a small group of 20 fibromyalgia patients. Varieties with high THC content significantly reduced the pressure pain threshold relative to placebo after a single inhalation. Interestingly, this effect was diminished by inhaling CBD, suggesting an antagonistic pharmacodynamics interaction of THC and CBD.^51^

A recent review of clinical trials of pain reduction by cannabis showed that cannabis-based medicines were most effective as adjuvant therapeutics in refractory multiple sclerosis and in managing chronic rheumatoid pain.^52^ Another group in New Zealand drew a similar conclusion after reviewing the literature on the usage of cannabis-based medicinal products for arthritis. They noted that while animal studies have shown a potential effect of cannabis products on arthritis pain, one randomized placebo-controlled study of Sativex® did not show an advantage over standard conventional pharmacological treatments. Therefore, they concluded that due to a lack of clear evidence, doctors should not be advised to prescribe cannabis-based medicines for arthritis.^53^ It is hypothesized that the analgesic activity of THC in chronic pain involves the function of two major cognitive-emotional modulating areas and their connections to somatosensory areas (summarized in Table 3).^54^

**Table 3:** Clinical Trials on Pain.

Reference	Research Goal	Study Design	Intervention	Participants *n* (F/M)	Cannabis Application	Tx Duration	Efficacy
Notcutt et al. (2004)^48^	Comparison of three CBME variants	Randomized, double-blind, placebo-controlled crossover trial	THC, CBD, THC+CBD, placebo	*n*=34	Sublingual spray	12 weeks	Highest efficacy of THC-based cannabis shown in pain regulation with only very mild side effects
Blake et al. (2006)^49^	Effectiveness of the CBME Sativex® on pain and morning stiffness	Randomized, double-blind, parallel-group trial	Sativex®, THC-based CBME McGill questionnaire	*n*=58: 31 CBME 27 placebo	Oromucosal spray	5 weeks	- Improvement in pain on movement and rest but no change in morning stiffness - No side effects or withdrawal signs
Wilsey et al. (2013)^50^	Short-term effects of CBME for neuropathic pain	Randomized, double-blind, placebo-controlled, crossover study	THC-based Medium dose (3.53%); low dose (1.29%) Placebo	*n*=35	Vaporizer 3–6 inhalations	300 min	- Cannabis had similar effects to conventional painkillers - Mild reversible psychoactive effects of limited duration
van de Donk et al. (2019)^51^	Comparison of four cannabis variants with known THC and CBD content	Randomized, placebo-controlled 4-way crossover study	Bedrocan* (22.4 mg THC, <1 mg CBD); Bediol* (13.4 mg THC, 17.8 mg CBD); Bedrolite* (18.4 mg CBD, <1 mg THC)	*n*=20 fibromyalgia patients	Vaporizer One single inhalation	3 hours	- No effect of treatments on spontaneous or electrical pain responses except 30% pain reduction in patients receiving Bediol - THC-containing varieties attained significant pressure pain reduction - Increment of THC plasma concentrations by CBD-containing varieties, but THC-induced analgesic effects were diminished
Haleem and Wright (2020)^52^	Effect of herbal cannabis and CBMEs on pain	Scoping review	Herbal cannabis and CBMEs	34 studies (30 RCTs, 4 non-RCTs)	Diverse	Diverse	- More promising results obtained from non-RCTs than from RCTs - Most promising results obtained for pain treatment as adjuvant therapy in refractory multiple sclerosis and rheumatoid pain

* Manufactured by Bedrocan International BV, Veendam, the Netherlands.

CBD, cannabidiol; CBME, cannabis-based medicinal extract; RCT(s), randomized controlled trial(s); THC, tetrahydrocannabinol.

### Retrospective Studies of Medicinal Cannabis Use

Retrospective studies in the form of exploratory cross-sectional surveys about recreational cannabis use among diagnosed rheumatology patients before and after cannabis legalization in Canada revealed that, after legalization, the percentage of cannabis users tripled from 4.3% to 12.6%. Half of the users had OA and used it for pain relief. Usually, the medicinal cannabis users were previous or current recreational users or with a history of drug abuse, younger than non-users, male, and of a low socioeconomic background. Different routes of application were used, ranging from smoking, vaporizing, and oral administration, and users lacked knowledge about product content. Only 20% of cannabis was acquired by the medicinal route, and only one-third reported marijuana use to their rheumatologist. Over 50% discontinued cannabis use because of lack of effect, and 28% due to adverse effects.^55^–^57^

Similar results were obtained in a retrospective nationwide survey from the United Kingdom, carried out from 1998 to 2002. A self-administered questionnaire about cannabis use completed by 2,969 participants revealed that medicinal cannabis was used for chronic pain (25%), multiple sclerosis and depression (22% each), arthritis (26%), and neuropathy (19%). Medicinal cannabis use was associated with younger age, male gender, and previous recreational use. The frequency of cannabis use was daily (35%), 3–5 days per week (23%), 1–2 days per week (15%), and less frequent (27%). The main administration route was smoking (82%), followed by eating (43%), drinking tea (28%), and other routes (14%). Symptom improvement was seen in 68% of users and a slight improvement by 27%. Users also stated that cannabis worked better (45%) or somewhat better (28%) compared to other medicines. Side effects compared to other medications were worse (6%), somewhat worse (23%), and the same (54%). After stopping cannabis intake, 77% of users stated that their symptoms returned or worsened. The authors concluded that this survey gave a broad picture of medicinal cannabis use and supported further development of safe and effective cannabis-based medicines.^58^

A recent meta-analysis came to a similar conclusion. Of 29,000 patients, 10,873 were cannabis users (40.4%), of which 15.3% were current users. A higher percentage of patients with fibromyalgia (68.2%), compared to 26.0% of patients with RA or lupus erythematosus, used cannabis. Cannabis users were younger of age (58.4% versus 63.6%), smokers (2.91% versus 1.84%), unemployed (2.4% versus 1.31%), and with higher pain intensity (5.0% versus 4.1%) compared to non-users. Cannabis consumption helped reduce the pain intensity on a VAS scale from 8.2 to 5.6. The meta-analysis concluded that about 20% of patients with rheumatoid diseases who actively consume cannabis report an improvement in pain (summarized in Table 4).^59^

**Table 4:** Retrospective Studies on Medicinal Cannabis Use.

Reference	Research Question/Goal	Study Design	Participants/Intervention		Outcome
			Control Group	Test Group	
Ware et al. (2005)^58^	Prevalence of medicinal cannabis users in the UK	Retrospective nationwide survey	2,969 participants		- MMJ used for: chronic pain (25%); multiple sclerosis (22%); depression (22%); arthritis (26%); neuropathy (19%) - Cannabis use associated with younger age, male gender, previous recreational use - Frequency of cannabis use: daily (35%); 3–5 days/week (23%); 1–2 days/week (15%); less frequent (27%) - Means of administration: smoking (82%); eating (43%); drinking tea (28%); other routes (15%) - Symptoms improvement: improvement (68%); light improvement (27%); cannabis worked better than other medicine (45%); cannabis worked somewhat better (28%) - Side effects of cannabis compared to other medications (6/872 worse; 23/872 somewhat worse; 4/782 about the same) - Side effects of other medications compared to cannabis (30% worse; 34% much worse; 26% impossible to tell)
Ste-Marie et al. (2016)^55^	Prevalence of marijuana users among rheumatology patients with confirmed diagnosis in Canada	Exploratory study and cross-sectional survey with coded questionnaires: (1) diagnosis entered by physician; (2) marijuana use entered by patient	MMJ non-users	MMJ users	- 4.3% were past MMJ users, of which 2.8% current users - No difference in disease prevalence between groups - MMJ users more likely to be on opioid treatment - Current MMJ users were younger (52.8 vs. 62.8 years), unemployed or disabled (46.4% vs. 7.9%), and tended to be male - MMJ users reported higher PtGA and pain scores than non-users (6.3% vs. 4.8%) - >80% of MMJ users reported previous recreational marijuana use - 80% of MMJ users were satisfied with the effects on pain relief, decreasing anxiety, nausea, and sleep improvement - Physician-assessed PGA did not differ between MMJ users vs. non-users - MMJ users had more severe disease than non-users, as measured by PGA and PtGA (patient-assessed) scores (≥6)
Jennings et al. (2019)^57^	Change in self-reported marijuana use in patients after the legalization of cannabis	Retrospective cohort study	*n*=500 before legalization (patients who had undergone arthroplasty)	*n*=500 after legalization (patients who had undergone arthroplasty)	- Self-reported use increased from 1% to 11% after legalization - Users after legalization: 46% recreational use, 26% medicinal use, 27% no reason, 2% recreational and medicinal use - Users were younger (10-year difference between users and non-users), male sex (61%), current smokers (37%), substance abuse history (14%), low socioeconomic background
Fitzcharles et al. (2020)^60^	Prevalence of marijuana users among rheumatology patients with confirmed diagnosis in Canada after marijuana legalization	Observational study, two questionnaires: (1) filled by the physician concerning diagnosis, (2) filled by patient concerning marijuana use	MMJ non-users	MMJ users	- <12.6% of rheumatology patients were current MMJ users - 28.3% were users of recreational cannabis, with 4.9% current users (of these, 44.9% currently used MMJ) - Current MMJ users were younger (61.2 vs. 64.9 years), unemployed or disabled (16.7% vs. 5.9%) - >80% of MMJ users reported obtaining cannabis via a non-medicinal route (personal contact, store, black market) - 69% of patients reported pain relief, 12% improved sleep, 15% improved fatigue, and 8% improved mood - PGA and PtGA scores for symptom relief: 6.7±2.5 - Adverse effects reported by 61.5% (35% cognitive effects, including drowsiness, fatigue, and lack of motivation; 26% anxiety; 20% lack of motivation; 26% more than one side effect)
Guillouard et al. (2021)^59^	Prevalence of cannabis use	Meta-analysis	*n*=18,127 non-users	*n*=10,873 cannabis users	Of 29,000 included participants, 40% had used cannabis, of which 15.3% were current users Use of cannabis for: - Fibromyalgia (68.2%) - RA or lupus erythematosus (26%) - Cannabis use associated with younger age, cigarette smokers, low socioeconomic background, higher pain intensity - Cannabis reduced pain intensity from 8.2 to 5.6 (VAS) - 20% of patients with rheumatoid diseases used cannabis and reported improvement in pain

MMJ, medical marijuana/cannabis; PGA, physician’s global assessment of disease activity; PtGA, patient’s global assessment of disease activity; RA, rheumatoid arthritis; VAS, visual analogue scale.

### Adverse Effects of Cannabis

Although adverse effects of cannabis-based medicinal extracts have been mainly described as mild and reversible, some studies have shown that patients consuming natural cannabis discontinued use due to side effects. These adverse effects mainly concern psychomotor and cognitive skills and the cardiovascular system.^61^ Psychomotor skill effects, including increased reaction time, disturbed selective attention, short-term memory, and motor control, are immediately affected by cannabis and can persist for up to 5 hours.^62^ The impact on cognition is seen as decreased learning abilities and retention of new information, and can last up to a few days. Moreover, driving ability and alertness are seriously impaired for up to 24 hours after herbal cannabis consumption,^63^ so it is not surprising that 0.5% to 7.6% of seriously injured drivers were found to be cannabis users.^64^

Severe cardiovascular events in connection with acute herbal cannabis use include tachycardia, hypotension,^65^ and an increased risk of myocardial infarction for people with angina pectoris.^66^ In a French Addictovigilance Network report, 35 vascular events were described between 2006 and 2010, with 26% leading to cardiovascular death.^67^

Regular cannabis use, especially in adolescents, might lead to a dose-dependent decline in cognitive performance and short-term memory, as well as mood disorders and even psychosis.^68^

The prevalence of medical cannabis use is steadily rising in the medical histories of individuals suffering from chronic pain. This global trend is exemplified, in part, by the fact that 40% of cancer patients turn to cannabis for pain relief in regions where medical cannabis is legally accessible, including countries like Canada, Germany, and Israel.^56^ Consequently, cancer patients may be at a higher risk of experiencing side effects and developing a dependence on cannabis.

With respect to cancer development, cannabis is often perceived as relatively benign, particularly in comparison to tobacco. However, recent research has revealed that smoking cannabis can lead to the production of carcinogens, such as nitrosamines and polycyclic aromatic hydrocarbons, which are akin to those found in cigarette smoke.^69^ Furthermore, cannabis smoke contains immunosuppressive agents and a mix of potentially mutagenic substances.^691^ Despite these discoveries, cannabis, unlike tobacco and alcohol, has not been conclusively established as a risk factor for cancer. Nonetheless, basic laboratory studies have demonstrated the mutagenic potential of cannabis *in vitro*.^70^

### Ongoing Clinical Trials

Only one ongoing interventional clinical trial was found in the National Library of Medicine’s database (ClinicalTrials.gov), conducted by Elizabeth Aston from Brown University, Providence, Rhode Island, United States. This double-blind, placebo-controlled, crossover study is currently recruiting 76 patients with psoriatic and rheumatoid arthritis to investigate the impact of cannabis on inflammation and pain. Cannabis with medium THC or medium CBD content will be administered via vaporization in two experimental sessions, and pain will be evaluated via self-reports. This phase 2 clinical trial will be the first study worldwide to examine the impact of two different cannabinoids in a clinical trial among patients with psoriatic arthritis or RA and may help develop a standard of care for the use of cannabinoids for arthritic treatment.^71^

Additionally, an observational study with 500 participants diagnosed with RA, spondyloarthritis, or psoriatic arthritis is examining the prevalence of cannabis use and aims to refine the characteristics of consumption and risk factors. This study hopes to further improve the overall management of patients with inflammatory rheumatic diseases.^72^

## CONCLUSION

**Figure 2:**
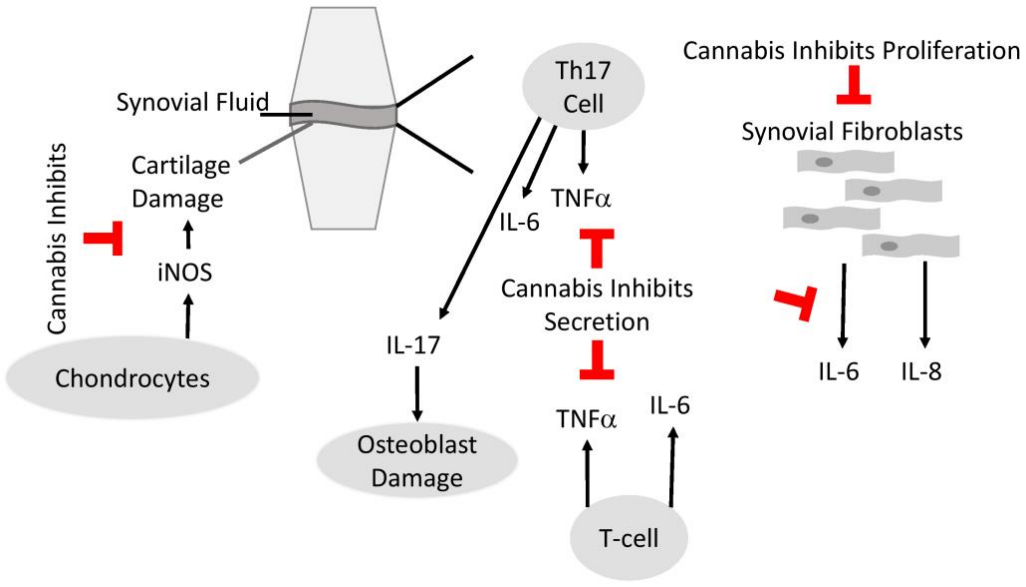
Anti-arthritic Properties of Cannabinoids. Scheme depicts the most important immune cells in the synovial fluid that contribute to the development of rheumatoid diseases and where cannabis has an anti-arthritic impact. Cannabis inhibits the proliferation of synovial fibroblasts, secretion of pro-inflammatory cytokines from immune cells, and the secretion of nitric oxide synthases, such as inducible NO synthase (iNOS), from chondrocytes, which prevents cartilage damage.

Preclinical *in vitro* and *in vivo* studies show promising results regarding the anti-arthritic properties of cannabinoids, psychoactive and non-psychoactive cannabinoids alike. These anti-arthritic properties are mediated by anti-inflammatory effects of cannabinoids, including inhibiting the production of pro-inflammatory cytokines and nitric oxide, as well as the proliferation of synovial fibroblasts (Figure 2).

These effects were primarily observed in preclinical *in vitro* and *ex vivo* studies as well as in animal models since clinical studies are scarce. One clinical study observed an increase in pro-inflammatory Th17 helper cells after the consumption of CBD oil in patients with RA. It was suggested that cannabinoid receptor variability might contribute to this discrepancy between preclinical animal and human results.^47^ Moreover, different cannabis strains can lead to different outcomes.^73^ Therefore, clinical studies that utilize well-defined cannabis strains will be able to target the outcome better and define the anti-arthritic properties of the administered cannabis strains.

Future research should focus on determining the exact anti-inflammatory properties of cannabis components for specific strains to more accurately provide targeted therapy to appropriate patients. This is one aspect of cannabis research that our research center is pursuing.

## Abbreviations

CB1R: cannabinoid receptor 1
CB2R: cannabinoid receptor 2
CBD: cannabidiol
DMH-11C: dimethylheptyl-THC-11-oic acid
DMARD(s): disease-modifying anti-rheumatic drugs
IL: interleukin
JAK/STAT: Janus kinase/signal transducer and activator of transcription
MMP(s): matrix metalloproteinase(s)
RNA: ribonucleic acid
miRNA: microRNA
NF: nuclear factor
OA: osteoarthritis
RA: rheumatoid arthritis
Th1: T helper 1
THC: tetrahydrocannabinol
TNF: tumor necrosis factor
TRP: transient receptor potential
